# Effects of child long-term illness on maternal employment: longitudinal findings from the UK Millennium Cohort Study

**DOI:** 10.1093/eurpub/ckw132

**Published:** 2016-08-26

**Authors:** Steven Hope, Anna Pearce, Margaret Whitehead, Catherine Law

**Affiliations:** 1 Population, Policy and Practice Programme, UCL Institute of Child Health, London, UK; 2 Department of Public Health and Policy, Institute of Psychology, Health and Society, University of Liverpool, Liverpool, UK

## Abstract

**Background:** Maternal employment has increased in European countries, but levels of employment are lower among mothers whose children have a limiting long-term illness or disability. However, we do not know whether having a child with a limiting illness prevents take-up or maintenance of paid employment or whether ‘common causes’, such as lack of qualifications or maternal disability lead to both maternal unemployment and childhood illness. Longitudinal data have the potential to distinguish between these. **Methods:** We analyzed four waves (3, 5, 7 and 11 years) of the Millennium Cohort Study (MCS) to examine the relationship between childhood limiting illness and maternal employment, unadjusted and adjusted for covariates. Multinomial regression models were used to test the association between child illness and trajectories of maternal employment. Fixed effects models assessed whether a new report of a child illness increased the odds of a mother exiting employment. **Results:** At every wave, maternal employment was more likely if the child did not have a limiting illness. After adjustment for covariates, childhood illness was associated with risks of continuous non-employment (adjusted Relative Risk Ratio = 1.46 [Confidence Interval: 1.21, 1.76]) or disrupted employment (aRRR = 1.26 [CI: 1.06, 1.49]), compared with entering or maintaining employment. If a child developed a limiting long-term illness, the likelihood of their mother exiting employment increased (adjusted Odds Ratio = 1.27 [CI: 1.05, 1.54]). **Conclusions:** ‘Common causes’ did not fully account for the association between child illness and maternal employment. Having a child with a limiting illness potentially reduces maternal employment opportunities.

## Introduction

Over recent decades, government policies in a number of European countries have promoted the uptake of paid work among parents as a route out of poverty.^1^ In UK, as in many other European countries, the number of mothers in employment has increased.^2^ However, as the primary carer in most families, mothers may have problems balancing the demands of caring for children and paid employment, particularly if a child has a long-term illness.^3^ Chronic health problems in children are associated with low maternal labour market participation. This association has been shown with a range of health conditions and definitions of poor health status.^3–8^

Two potential mechanisms through which an association between childhood long-term limiting illness and low maternal labour market participation might arise are considered. First, there is ‘common cause’, where socio-demographic and maternal health characteristics increase risks of both childhood illness and maternal non-employment.^9^^,^^10^ The relationship between childhood illness and maternal employment status is likely to be complicated by the fact that socio-economic disadvantage is associated with both disability in childhood^11^ and non-employment;^12^ and maternal disability predicts both employment status^12^ and child health problems.^13^ Second, having a child with a limiting illness may restrict maternal employment opportunities or precipitate departure from the labour market directly, as a consequence of the complex caring responsibilities required when raising a child with a chronic health condition.^14^ Children with a long-term illness are likely to have additional needs compared with other children,^5^ which may in turn create difficulties for parents who wish to enter the labour market or remain in employment.^8^

Most studies investigating the relationship between childhood illness and maternal employment have used small, cross-sectional or non-representative samples, providing little opportunity to properly investigate mechanisms.^5^ Although there has been some longitudinal research,^3^,^15–17^ there is very little European evidence.^9^^,^^10^^,^^18^ In order to investigate these two potential mechanisms, we used longitudinal data from Millennium Cohort Study, a large, contemporary cohort of UK children born in 2000, with information on childhood long-term limiting illness and maternal employment recorded in early to middle childhood. We investigated the extent to which: (i) mothers of children with a limiting long-term illness were less likely to enter or maintain undisrupted employment, after taking account of potential socio-demographic and health covariates indicative of a ‘common cause’ and (ii) the development of a childhood limiting long-term illness is associated with an increased likelihood of employed mothers leaving the labour market.

## Methods

### Subjects and design

We examined data from the Millennium Cohort Study (MCS), a longitudinal study of children born in UK in 2000–2002.^19^ Survey interviews were carried out in the home with the main respondent (almost always the mother). Our analyses used data from successive data collection waves when the child was aged 3, 5, 7 and 11 years. The families of 15 381 singleton children were interviewed when the child was 3 years of age. Attrition is a problem common to all cohort studies, and by age 11 years the number of families who had participated in all subsequent waves had declined to 10 702 (70% of the sample at 3 years).

Further exclusions were made when the main respondent was someone other than the mother at one or more waves (in order to investigate maternal employment status and to have a consistent rater of child limiting illness) reducing the sample to 9973. Of these families, only those with complete data on child limiting illness status (9831) and maternal employment (9747) were retained. In logistic regression models including time-invariant and time-varying covariates recorded at the 3 year wave, those with missing data on covariates were excluded, reducing the sample to 9634. In fixed effects models, full data were required for time-varying covariates between 3 and 11 years, reducing the sample to 9636 children. The analytic sample for fixed effects analyses was based on 4004 children where there was a change in maternal employment status during the course of the study. Data were obtained from the UK Data Archive, University of Essex on 14th February 2014.

### Limiting long-term illness

At ages 3, 5, 7 and 11 years, mothers reported whether their child had any long-term conditions that limited their ability to perform daily activities. A binary measure was derived based on whether the child had a reported limiting long-term illness at any wave between ages 3–11 years.

### Maternal employment

Mothers of the children reported their current employment status at each of the data collection waves. Cross-sectionally, from 3 to 11 years, the risk of not being in employment (compared with being employed) was greater for mothers of children with a limiting illness. However, there were no differences according to intensity of employment (full-time vs. part-time) and so full-time and part-time were combined into a single employed group for further analyses.

In order to examine stability and change in employment status during the period when the child was between 3 and 11 years of age, three summary trajectories for maternal employment were derived: those who entered or maintained employment, those who experienced disruption (characterised by at least one transition out of employment) and those who were not employed at any wave.

### ‘Common cause’ factors

To account for ‘common cause’, or confounding, factors for both child limiting long-term illness and maternal employment, we adjusted for a number of covariates. Time-invariant maternal characteristics were captured at baseline: mothers’ highest qualification and age at the birth of the cohort child. Other factors that were more likely to vary between waves were: maternal long-term illness, and household characteristics (lone parent family, number of children in the household). In multinomial logistic regression analyses, we adjusted for these at 3 years of age. As a sensitivity analysis, we repeated these analyses adjusting for covariates at 11 years. There was no difference in the pattern of results, and so only those for covariates at 3 years are presented.

### Statistical analysis

All analyses were conducted in Stata/SE 13 (Stata Corporation, TX), ‘svy’ commands to allow for clustered sampling design and attrition up to 11 years. Weighted percentages (using survey and non-response weights) and 95% confidence intervals (CIs) of childhood limiting illness and covariates were calculated according to employment trajectories. We used logistic regression to estimate odds ratios (ORs) and 95% CIs for non-employment by childhood limiting illness at each wave.

Multinomial logistic regression models were used to estimate Relative Risk Ratios (RRRs) and 95% CIs for employment disruption or continuous non-employment between ages 3–11 years (baseline continuous employment or entering employment), according to any occurrence of childhood limiting illness across the same period. Analyses were conducted unadjusted and adjusted for potential ‘common cause’ covariates at baseline.

We also tested whether the development of a childhood limiting illness was associated with a subsequent labour market exit. We used fixed effects models to estimate ORs and 95% CIs for exiting employment associated with a change in child limiting illness, unadjusted and adjusted for time-varying covariates (maternal lone parent status, long-term illness and number of children in the household).

## Results

### Cross-sectional analyses

The prevalence of reported child limiting long-term illness increased from 3% (284) at 3 years to 8% (700) at 11 years. Over the same period, maternal employment increased from 49% (5036) to 67% (6691). However, at every age, mothers of children with a limiting illness were less likely to be employed than other mothers. This discrepancy in proportion of mothers in employment increased with every wave, from 3 years (no child limiting illness: 50% mothers employed vs. child limiting illness: 39% mothers employed) to 11 years (no child limiting illness: 69% employed vs. child limiting illness: 53% employed), with an increase in the odds of non-employment associated with child limiting illness from OR = 1.53 [CI: 1.17, 2.01] at 3 years to OR = 1.91 [CI: 1.59, 2.30] at 11 years.

### Continuous non-employment

The majority of mothers entered or maintained employment (59%), while similar proportions either experienced disruption (20%) or never entered paid employment (21%) during the study period. Compared with mothers who entered or maintained employment, those who never entered employment had a higher prevalence of having a child with a limiting illness between 3 and 11 years (table 1). They were also more likely to have a long-term illness themselves, to have three or more children in their household, to live in a lone parent household, to have been younger at the birth of the cohort child and to have lower or no educational qualifications. In a multinomial model, with mothers who entered or maintained employment as the baseline group (table 2), those who reported a child with a limiting long-term illness had a raised risk of never being employed. Adjusting for ‘common cause’ factors attenuated this association, but it remained elevated (RRR = 1.46 [CI: 1.21, 1.76]).

**Table 1:** Prevalence of child limiting long-term illness and covariates, according to maternal employment trajectories when child aged between 3 and 11 years (*n* = 9634)

	Entered or maintained employment (*n* = 5892)	Experienced disrupted employment (*n* = 1830)	Never entered employment (*n* = 1912)
	Percent [95% CIs]	Percent [95% CIs]	Percent [95% CIs]
**Child limiting long-term illness reported at 3, 5, 7 or 11 years**			
Yes	12.6	16.6	20.3
	[11.6, 13.7]	[14.6, 18.8]	[17.9, 22.8]
***Covariates***			
**Maternal long-term illness at 3 years**			
Yes	18.3	24.0	28.6
	[17.2, 19.5]	[21.6, 26.4]	[26.1, 31.2]
**Number of children in household at 3 years**			
1 child	23.6	32.4	16.8
	[22.3, 25.0]	[29.9, 34.9]	[14.6, 19.3]
2 children	51.5	44.2	39.7
	[49.9, 53.0]	[41.5, 46.9]	[37.0, 42.6]
3 or more children	24.9	23.5	43.5
	[23.5, 26.4]	[21.3, 25.7]	[40.7, 46.2]
**Lone parent household at 3 years**			
Yes	12.5	17.0	29.4
	[11.3, 13.8]	[14.9, 19.4]	[26.5, 32.5]
**Maternal age at birth of child**			
14–19 years	4.4	9.7	14.3
	[3.7, 5.2]	[8.0, 11.7]	[12.0, 16.9]
20–29 years	40.3	52.1	52.3
	[38.2, 42.4]	[49.0, 55.1]	[49.0, 55.5]
30–39 years	52.9	35.8	31.3
	[50.8, 55.0]	[32.6, 39.2]	[28.5, 34.3]
40+ years	2.4	2.4	2.2
	[2.0, 3.0]	[1.7, 3.4]	[1.6, 3.0]
**Maternal highest qualification at 3 years**			
A level/degree (high educational level)	45.8	36.7	19.9
	[43.3, 48.4]	[33.2, 40.1]	[17.1, 23.2]
O level/GCSEs (middle educational level)	46.4	49.3	45.0
	[44.5, 48.8]	[46.2, 52.4]	[41.9, 48.1]
No qualifications (low educational level)	7.8	14.0	35.2
	[6.8, 8.8]	[12.1, 16.3]	[31.7, 38.6]

**Table 2:** Relative risk ratios [95% CIs] for disrupted employment or never entering employment, according to child limiting long-term illness (3, 5, 7 or 11 years) (*n* = 9634)

	Entered or maintained employment	Experienced disrupted employment	Never entered employment
***Unadjusted***			
No limiting long-term illness	–	–	–
Child limiting long-term illness	1.00	1.38	1.76
		[1.18, 1.61]	[1.48, 2.09]
***Adjusted for covariates***^a^			
No limiting long-term illness	–	–	–
Child limiting long-term illness (adjusted for covariates^a^)	1.00	1.26	1.46
		[1.06, 1.49]	[1.21, 1.76]

a: Mothers’ age at cohort child birth, maternal highest qualification, maternal long-term illness, number of children in household (all measured at baseline).

### Disrupted employment

Prevalence of child limiting illness between 3 and 11 years was also greater among mothers who had experienced disrupted employment during this period, compared with those who entered or maintained employment (table 1). The ‘common cause’ factors reported by mothers with disrupted employment histories tended to fall between those of the other two employment trajectory groups, with a greater likelihood of possessing characteristics indicative of ill health and lower socio-economic circumstances than those who entered or maintained employment, but a lower likelihood than those who had never been employed. Differences between the disrupted and never employed trajectories were greatest for number of children in the household (three or more), lone parent status, maternal age at birth (14–19 years) and highest qualification achieved (no qualifications). In the multinomial model (table 2) there was a significantly elevated risk of disrupted maternal employment among those who had a child with a limiting illness, which remained after adjusting for the covariates (RRR = 1.26 [CI: 1.06, 1.49]).

### Impact of the development of child limiting long-term illness on leaving the labour market

The previous analysis identified that child limiting illness occurring at any point throughout early to middle childhood was associated with disrupted employment over the same period. We also used fixed effects models to examine whether the reporting of a new limiting illness in a cohort child between two data collection points was associated with an increased risk of exiting employment over the same two points in time. If a child developed a limiting illness between waves, the odds of their mother exiting employment was increased (OR = 1.28 [CI: 1.06, 1.54]) compared with children who did not develop a limiting illness. This result was not attenuated by adjustment for time-varying covariates: maternal long-term illness, number of children in the household and lone parent status (OR = 1.27 [CI: 1.05, 1.54]).

## Discussion

Using a national cohort of UK children followed from 3 to 11 years, we found evidence of an association between childhood limiting long-term illness and maternal labour market participation. Levels of maternal employment increased over time as children in the sample entered middle childhood. During the same period, prevalence of limiting illness in the cohort children also increased. Levels of employment did not increase to the same extent for mothers in families containing a child with a limiting illness.

These results are in line with the existing literature, much of which is cross-sectional.^5^ However, we hypothesised two potential causal pathways between childhood illness and maternal employment. First, ‘common cause’ posits that there would be common socio-demographic and health predictors of both childhood illness and maternal employment. Mothers who were never employed or who exited employed were more likely to be socio-economically disadvantaged or to report health problems than those who were continuously employed or entered and remained in employment. Our results, however, showed that the relationship between childhood illness and maternal employment remained, although attenuated, after adjustment for the covariates, which indicates that ‘common cause’ does not offer a full explanation. Nevertheless, it is possible that other, unmeasured, ‘common cause’ factors may underlie these results.

Second, we proposed that having a child with a limiting illness may restrict maternal employment opportunities or precipitate a departure from the labour market. Our findings support this hypothesis. We found that mothers who reported that their child had a limiting illness at one or more data collection waves were at an increased risk of never having been employed or having had an unstable employment history during this period. In addition, results from fixed effects models showed that the development of a limiting illness was associated with mothers exiting employment. These findings suggest that having a child with a limiting illness may reduce opportunities both for mothers to maintain employment and, for non-employed mothers, to seek employment.

This study is novel in providing European evidence on the relationship between childhood illness and maternal employment among children progressing from early to middle childhood. A particular strength of our study is the use of the large, UK-representative MCS, which allowed us to follow children over time and to distinguish between the two competing mechanisms. However, most longitudinal studies, including the MCS, are subject to attrition and missing data, which may lead to bias. The majority of cohort children with a limiting illness at 3 years of age, participated in all waves of the MCS (65%), although this proportion was smaller than was the case when there was no limiting illness (70%). A similar pattern of differential attrition was observed for limiting illness reported at subsequent waves, and we may therefore have underestimated the proportion of children with a limiting illness in the MCS.

We assessed whether differential attrition and missingness may have influenced the key relationship in the paper, between child limiting illness and maternal employment in a number of ways. Firstly, we compared the prevalence of maternal employment, and its association with limiting illness, cross-sectionally at each wave (3, 5, 7 and 11 years) in the analytic sample vs. cross-sectional samples (where the only restriction was that the mother was the main respondent at that wave). We found that the prevalence of maternal employment at each wave was higher in the analytic sample. However, the difference between the proportions of mothers employed when a child had a limiting illness compared with when they did not was almost identical at each wave in the analytic and cross-sectional samples. These findings suggest that the relationship between limiting illness and maternal employment is robust. Nevertheless, the possibility exists that bias due to non-participation and missing data may have occurred, although we used response weights to account for attrition up to the 11-year survey.^20^

Despite the size of the study, there were relatively few children with a limiting illness at any data collection wave. The measure of illness was reported by the mother, and the question wording is similar to that used to measure long-standing illness in UK population surveys, such as the Health Survey for England. The measure of illness was reported by the mother, and the question wording is similar to that used to measure long-standing illness in UK population surveys, such as the Health Survey for England. These measures have been widely used in research on childhood chronic illness or disability, and prevalences during childhood shown here are comparable to those published elsewhere.^21^^,^^22^ Report of limiting illness has been found to be a valid indicator of morbidity in a range of age groups^24^. Nevertheless, generic questions on limiting long-standing conditions do not differentiate diagnosis, and will reflect the mothers’ interpretations of both illness and limitation. When probed by interviewers about the underlying health problem, a large number of varied conditions (either individually or comorbid) were reported by mothers, to the extent that it would be impossible to investigate pathways between specific diagnoses and maternal employment despite the large sample size in the MCS. However, our aim was not to focus on the potentially diverse mechanisms by which particular diagnoses may impact on maternal employment, but, rather, on whether a child’s health limitations (regardless of underlying health condition or conditions) are associated with maternal employment status. The questions on limiting illness in the MCS were suitable for this, providing an opportunity for mothers to indicate the extent to which they perceived their child’s health conditions affected everyday activities.

Employment status was based on that currently reported at each of the waves, and so will have overlooked fluctuations in employment status occurring between waves. However, these data provide insights into the working lives of mothers, and will be less prone to the recall bias likely to result from retrospective employment histories. While a more detailed analysis of the characteristics and context of employment was beyond the scope of these analyses, it should be an area for future research.

### Implications for research and policy

More research is needed to reveal whether these mothers made a decision to take on a caring role for their ill child themselves, with consequences for labour market participation, or whether they would have wished to have employment. However, there is evidence that a large proportion of mothers of children with disabilities have a strong desire to enter employment,^23^ with issues such as childcare and service limitations mentioned as constraining factors for entering employment.^14^ For mothers caring for a child with a chronic health problem, employment is less likely to be permanent or secure.^17^ In terms of policy implications, the findings suggest that there needs to be further consideration of how to effectively support the employment decisions of parents of children with a long-standing illness, such as providing greater opportunities for flexible working and parental leave. However, implications extend beyond employment and social security, to policies on support for parents’ caring roles, including improved availability of affordable childcare suitable for children with a disability or chronic health problem, and short-term flexibility when children are acutely ill or need to attend health services.

## Funding

This work was supported by the Public Health Research Consortium (PHRC). The PHRC is funded by the Department of Health Policy Research Programme. The views expressed in the publication are those of the authors and not necessarily those of the Department of Health. Information about the wider programme of the PHRC is available from http://phrc.lshtm.ac.uk/. A.P. is funded by a Medical Research Council Population Health Scientist Fellowship (MR/J012351/1). The Population, Policy and Practice Programme was formed in 2014, incorporating the activities of the Centre for Paediatric Epidemiology and Biostatistics (CPEB). The CPEB was supported in part by the Medical Research Council in its capacity as the MRC Centre of Epidemiology for Child Health (award G0400546). Research at the UCL Institute of Child Health and Great Ormond Street Hospital for Children receives a proportion of the funding from the Department of Health’s National Institute for Health Research Biomedical Research Centres funding scheme. The Millennium Cohort Study is funded by grants to former and current directors of the study from the Economic and Social Research Council (Professor Heather Joshi, Professor Lucinda Platt and Professor Emla Fitzsimons) and a consortium of government funders. The study sponsors played no part in the design, data analysis and interpretation of this study; the writing of the article or the decision to submit the paper for publication, and the authors’ work was independent of their funders.

## Disclaimer

The views expressed in the publication are those of the authors and not necessarily those of the Department of Health.

*Conflicts of interest*: None declared.

Key points

- We used longitudinal data to examine the effects of child limiting long-term illness on maternal employment, testing whether any relationship could be explained by ‘common cause’ factors vs. the direct impacts caring responsibilities may have on mothers’ employment opportunities.
- Childhood illness was associated with non-employment and disrupted employment, after adjusting for covariates, suggesting that a ‘common cause’ hypothesis does not offer a full explanation.
- The findings suggest that there needs to be consideration of how to support the employment decisions of parents of children with a chronic health problem or disability.
